# Informal social accountability in maternal health service delivery: A study in Northern Malawi

**DOI:** 10.1371/journal.pone.0195671

**Published:** 2018-04-11

**Authors:** Elsbet Lodenstein, Christine Ingemann, Joyce M. Molenaar, Marjolein Dieleman, Jacqueline E. W. Broerse

**Affiliations:** 1 Athena Institute for Research on Innovation and Communication in Health and Life Sciences, Faculty of Earth and Life Sciences, VU University, Amsterdam, The Netherlands; 2 KIT Gender, Royal Tropical Institute, Amsterdam, The Netherlands; 3 KIT Health, Royal Tropical Institute, Amsterdam, The Netherlands; University of North Carolina at Chapel Hill, UNITED STATES

## Abstract

Despite the expansion of literature on social accountability in low-and middle-income countries, little is known about how health providers experience daily social pressure and citizen feedback. This study used a narrative inquiry approach to explore the function of daily social accountability relations among maternal health care workers in rural Malawi. Through semi-structured interviews with 32 nurses and 19 clinicians, we collected 155 feedback cases allowing the identification of four main strategies social actors use to express their opinion and concerns about maternal health services. We found that women who used delivery care express their appreciation for successful deliveries directly to the health worker but complaints, such as on absenteeism and poor interpersonal behaviour, follow an indirect route via intermediaries such as the health workers’ spouse, co-workers or the health committee who forward some cases of misbehaviour to district authorities. The findings suggest that citizen feedback is important for the socialization, motivation and retention of maternal healthcare workers in under resourced rural settings. Practitioners and external development programmes should understand and recognize the value of already existing accountability mechanisms and foster social accountability approaches that allow communities as well as health workers to challenge the systemic obstacles to quality and respectful service delivery.

## Introduction

Responsive, people-centred and respectful maternal health care is increasingly recognized as a critical component of efforts to improve maternal health outcomes and reduce maternal mortality [1–3]. In low-and middle-income countries, investments in human resources for health, facility infrastructure and technology are considered crucial to support and sustain respectful care [4]. Other strategies, more related to the governance of health systems, include strengthening accountability, supervision and regulation, the use of ethical codes of conduct and community-level awareness of maternal health rights [3,5,6]. This paper focuses on the role of social accountability in influencing maternal healthcare workers’ (HW) practices.

In general terms, social accountability refers to citizens’ demands for greater accountability from political and governmental actors for their actions and decisions, as well as for service delivery failures [7]. Translated to the level of frontline service provision, social accountability may involve the monitoring of health services by the public and the use of feedback and complaint mechanisms to address failures in service delivery. Through a process of assessment, demand articulation, feedback and negotiation with providers, changes in provider behaviour and facility practices are expected [7,8]. Over the past few decades, civil society organisations, often with international development support, have sought to facilitate these processes by organising and structuring procedures for citizen monitoring and feedback and community-provider dialogue, for example through social audits or community score cards [9].

Evidence on the effect of such interventions is mixed because data on interventions in different contexts is fragmented [10]. Social accountability scholars have argued that the complex nature of accountability relations calls for more political and context-sensitive approaches to study social accountability [7,11]. They urge for studies that help to understand social accountability as an ongoing and already existing dynamic of interaction between social actors and the state, rather than as individual interventions or tools [9]. Social accountability is increasingly understood as part of an accountability “ecosystem” [12], “culture of accountability” [13] or “micro-network” of accountability relations [14]. In such networks, service users, citizens or other social actors call health providers to account in formal and organized forums but also in informal, spontaneous and unorganized ways. The informal forms of social accountability are considered particularly relevant in contexts where formal direct accountability mechanisms, such as official complaint mechanisms, public ombudsmen, participatory planning and monitoring, and local management committees, are absent or not enforced [15,16]. Informal social accountability, however, has been least documented. Two case studies are often cited: Hossain (2008) discusses forms of “rude accountability” in Bangladesh referring to citizens reacting in unorganized, spontaneous and sometimes violent ways to service failures [16], while Tsai presents a study from China in which citizens use local solidarity groups to pressure for change [17]. This study attempts to contribute to the scholarship on social accountability, in particular its’ informal expressions, by investigating the ways in which maternal health care providers are held to account by social actors in daily practice in Northern Malawi.

Studies on the health system in Malawi sketch a grim picture of the accountability landscape in which citizens have a limited “repertoire” of social action [13] and in which rural health providers are not affected or reached by formal accountability mechanisms. On the demand side of social accountability, a study on the perceptions of care and accountability in Malawi by Jones *et al*. (2013) showed that even though health service users are aware of their rights and expectations in healthcare, they lack effective channels through which they can voice their concerns and complaints and hold health professionals accountable [18]. Health Centre Advisory Committees (HCACs) constitute the formal channel for user input into local service delivery, but they are hampered by a lack of skills and resources to perform their tasks [19]. Beyond the HCACs, citizens rarely approach other representatives such as local councillors, political leaders or government officials to give their view or report issues [20]. Local courts are inaccessible for rural people and many communities lack trust in these institutions [21]. In order to promote citizen voice in service delivery and address the above mentioned accountability failures, non-governmental organisations (NGOs) support several initiatives in Malawi, such as Citizen Hearings and Community Score Cards in maternal health service delivery. While these initiatives have triggered attention to service delivery failures, including in the media, national implementation has been uneven and challenges remain in linking such initiatives to the wider health system [22,23]. On the supply side of accountability, formal accountability measures within the health system are considered weak. Monitoring and supervision by district personnel is often not conducted as required, one of the reasons being the high costs and the lack of qualified supervisors [24,25]. A study by Bradley *et al*. (2013) in Malawi found that many rural facilities are monitored infrequently by District Health Management Teams, while another study reports that 28.7% of health workers in Malawi receive no supervision at all [26,27]. The lack of supervision and peer support leaves rural health workers feeling abandoned and remote from the government [26,27].

In this context, we assume that the provision of respectful services may rely, to a large extent, on local accountability arrangements between citizens and providers. Formal participatory mechanisms, such as Health Committees, may be part of these arrangements, but informal networks and relationships may be just as important [28]. This paper presents an analysis of the role of informal social accountability relations in monitoring and promoting respectful maternal healthcare in Northern Malawi. Such analysis helps to develop locally relevant social accountability interventions and to identify potential avenues for strengthening linkages between informal and formal accountability mechanisms in the health system in Malawi.

### Conceptualising informal social accountability

In this paper, we use elements of the conceptual accountability framework by Bovens [29]. We refer to social accountability as a practice of account giving by an “actor”, in our case a health worker, to a “forum”, in our case civic or social actors, defined as individuals or groups in the community in the external or non-work environment of health providers and the health facility. The forum includes groups of service users, citizens, traditional institutions, such as chiefs in Malawi, and community representatives in the HCAC, but excludes co-workers, managers and community health workers and, at a district level, supervisors and other government representatives.

The accountability relationship between the actor and the forum consists of several steps including the *information* of the forum about the responsibilities standards and outcomes of the conduct of the actor followed by the *questioning*, *discussion* and *passing of a judgment* of the actor’s conduct. Finally, the forum can impose *consequences* to the actor; rewards for a positive, and sanctions for a negative judgement [29]. In common with Bovens (2007), we posit that accountability is primarily retrospective. Existing literature provides some ideas on what informality means in this process.

Firstly, standards and norms of good maternal health care are likely constructed locally and assessment and judgement are likely to be based more on social norms, values and expectations than on predefined quality standards [30]. Furthermore, social accountability is not necessarily exercised in formal organised or “invited” [31] spaces such as Health Committees. At the “street-level” of frontline service provision, it can also be exercised in interpersonal interactions between patients and service providers [14]. Any individual, group or organisation can initiate or facilitate an interaction or forum to pose questions, provide feedback and impose consequences on health workers [29]. Also, in informal social accountability, feedback from social actors to health providers is likely to be ad-hoc (non-repeated), unofficial, informal, personalized, and “organic” [16]. It can be passive (unsolicited information), active (engaging health providers), verbal, non-verbal and written [32]. Finally, the possibility of formal sanction for poor performance is often limited for citizens, as health providers are often not obliged to report to a citizen forum [29]. Rather than relying on authoritative power and sanctions, social actors use soft power or “soft pressure”, namely the ability to *persuade* through insistence on collective values rather than to *coerce* through “hard” sanctions, threats or financial incentives [7,14,33]. Persuasion, then, relies on alternative sources of power, such as knowledge and information, experience and commitment, representation and solidarity [7,34] and sanctions can include a range of expressions and actions, such as admiration or contempt, approval or disapproval, praise or rebuke, compliment or insult, promises of physical reward or threats of physical punishment, and actual physical reward or punishment [35]. This framework has informed the data collection and analysis tools.

## Methods

### Research context

The study was conducted in the Northern region of Malawi in one of the six districts, Mzimba district, between April and June 2016. The Northern Region is the least populated region, home to only 13% of the total population [36]. The district was purposefully selected; the study was carried out as part of a larger study in partnership between VU University, an NGO and district authorities that aimed to strengthen the quality of maternal health care.

Primary health care in Malawi is offered by a network of around 977 functional health facilities, including health posts, health centres (HCs) and hospitals. These comprise public-governmental facilities (50%) as well as private non-for-profit facilities of the Christian Health Association of Malawi (CHAM) (17%) and other providers (33%) [36]. HCs, the health facilities of interest to our study, have a catchment area between 5,000–20,000 people. Most HCs provide a basic package of services (child care, vaccination, family planning, antenatal and normal delivery care, STI) while only 37% are on-duty or on-call 24 hours per day [37], particularly burdensome for emergencies and delivery care. The proportion of births assisted by skilled health personnel in the Northern Region is reported to be 90.6% in 2015 [38]. Maternal healthcare, as most other care, is provided free of user fees.

This study targeted rural HCs and hospitals in Mzimba district, both government and CHAM owned. The government also employs health staff in the CHAM HCs and the working conditions in both types of HCs are comparable. A total of 39 HCs in Mzimba district were eligible for the study as rural facilities providing maternal health care. Administratively, the district is divided in two subdistricts, Mzimba North and South, containing respectively 12 and 27 eligible HCs. Each subdistrict is further divided into clusters of HCs that are constructed by the District Health Management Team for their supervision visits on the basis of the geographical concentration of HCs. Subdistrict North contained one cluster covering all the 12 HCs that were selected for the study and subdistrict South contained six clusters of which three clusters were selected based on a random sampling strategy. The selected three clusters in the south hosted a total of 14 HCs. Hence, the total number of HCs included in the study was 26, of which six were CHAM and 20 government owned. Their distance to the district capital (and district hospital) ranged from 15 to 132 kilometres. Nine of the selected facilities had three or more skilled birth attendants, 13 had two skilled birth attendants, and four had just one. The number of deliveries conducted ranged from 36 to 723 in the year 2014 [39]. In most sites, traditional chiefs represent the local authority while the HCAC represents the formal structure related to the HC. In terms of social accountability interventions, to the knowledge of the District Health Officer and key informants, some HCACs had been trained on drug monitoring and complaint management. Radio programmes occasionally discuss the quality of service delivery in the district.

### Selection and recruitment of participants

From the 26 selected HCs, a purposive sampling of the study population was based on the following eligibility criteria: 1) government employed skilled birth attendants (doctors, clinical officers, medical assistants, nurses and midwives)[40], identified as ‘health workers’ (HWs), 2) performance of basic or emergency obstetric care within the last three months, 3) worked in the health facility for at least four months, and 4) available without disruption or interruption of the health service delivery at the time of data collection. The district health offices in Mzimba North and South provided information and contact details on the HWs. These details assisted in establishing travel schedules, making appointments in advance and announcing the visit prior to arrival. All selected and invited HWs were available and agreed to participate in the interviews.

Table 1 presents the participants’ characteristics. A total of 51 maternal HWs were included. Almost two thirds were nurses (63%) and one-third clinicians (37%). Twenty-eight participants were male (55%), but the category of nurses was dominated by female participants (66%); both distributions are representative of the Malawian health workforce [41]. Participants worked in rural HCs (90%) and in rural hospitals (10%). The majority of HWs were employed by the Ministry of Health (80%), the others by CHAM (20%). Thirty-five HWs had worked in the study facility for less than three years and 16 had worked there longer than three years of which 12 (75%) were nurses. The majority of HWs (41%) were younger than 30 years, while 29% were between 30 and 40 years of age. Thirty-eight (75%) HWs reported that they assist in childbirth at least twice a week, with just over half (52%) on a daily basis.

**Table 1:** Participants by profession and gender.

Category of maternal health care worker	Female	Male	Total
Clinician	2	17	19 (37%)
Nurse	21	11	32 (36%)
	23 (45%)	28 (55%)	51

### Data collection

The main data collection method was individual, semi-structured interview with nurses and clinicians. The interviews were divided between two researchers who each conducted around 25 interviews within a period of five weeks. The main goal of the interview was to gather information on social accountability relations and processes. The technique chosen was narrative inquiry, a research method that collects personal experiences of past events [42]. A scoping study in Malawi and test interviews supported the contextualization of our conceptual framework. It appeared that “feedback” was a commonly known and used term by HWs as well as district health authorities and mostly referred to verbal interactions–also in terms of feedback after supervision visits. We used the concept of “feedback” to refer to the actual interaction process between social actors and HWs. Adapted from a definition of feedback in clinical settings by Jamtvedt *et al*. (2010), feedback is defined as a statement or a summary of performance of delivery care over a specified period of time or regarding one event (i.e. assisted delivery) [32]. We developed three key questions to collect narratives of social accountability: 1) “How do people let you know what they think of your services?”, 2) “Could you tell me of a time when you felt appreciated by the community for the services you provided?’, and 3) “Could you tell me of a time you felt rejected by the community for the services you provided?”. Respondents were asked to recall incidents in the past two months.

The interviews were conducted in English and lasted approximately 50–60 minutes. The three main questions were preceded by a mapping exercise of social actors in the immediate environment of the respondent. The actors enumerated were displayed in front of the respondent after which the three questions were posed. For each case presented, respondents were probed to explain when and how the situation occurred. The narrative inquiry approach, alternating engaging and sensitive questions, was expected to yield data that could show the breadth of HWs’ web of social accountability relations, including cases of criticism that might be difficult to share.

The individual interviews were complemented by two group discussions (one in each district) with HWs who participated in the study (n = 12) and new participants (n = 3). The aim was to share and validate the preliminary findings of the interviews, to evaluate the research and to discuss practical implications.

### Data analysis

The interviews and group discussions were audio recorded, except for one interview where the respondent did not want to be recorded. The audio recordings were transcribed verbatim by the researchers and externally employed transcribers from Malawi. Sensitive data was removed from the transcripts.

The interviews led to narratives of social accountability that we framed as “feedback cases”. Cases were extracted from the transcripts with the help of qualitative data analysis software, MaxQDA (version 12), and coded using framework analysis. This method allows contrasting and comparing data by themes across many cases, as well as retaining the connection to other aspects of individual accounts [43]. For each case, data on the *what* (was the feedback about?), *who* (were the providers/messengers of feedback?) *when* and *where* (did it happen?), *how* (was the feedback transferred?) were coded, as well as the perception of the respondent on the case (feeling, influence on work). Duplicate cases, where a respondent mentioned the same case twice in an interview, were removed as well as cases that were not clear in terms of the main codes: topic, social actor, or feedback method. The codes helped to get a first overview of frequencies of these main codes. In a next stage, a cross-case analysis was performed to identify patterns, every time taking a different code as entry point (e.g. social actor or type of feedback), to identify the theme that made most analytic sense. This was done in an iterative way through inter-researcher reflection, reference to notes and group discussion notes, and discussion among researchers. We used standard frequency analyses in SPSS (version 22.0) to describe HW characteristics.

### Ethical considerations

Ethical approval for this study was granted by the National Health Science Research Committee of the Ministry of Health in Malawi (NHSRC#15/03/1398). Prior to the interviews and group discussions, the researchers introduced themselves and the research project, read the informed consent form out loud, and participants were asked to provide written or verbal consent. Consent to audiotape the interview and discussions was obtained through informed consent as well as prior to the start of the interview by asking the participants. All information given by participants during the research was considered to be strictly confidential and the findings of the research were based on anonymized data as described in the section above regarding data management (48). Moreover, transcribers for this research were selected based on the criteria that they did not have any links to the communities and HCs included in the study in order to ensure anonymity [44]. The transcribers have also been instructed in and signed a form to ensure the duty of professional confidentiality.

## Results

In total, 155 feedback cases were collected. Respondents were allowed to share more than one example of a feedback case. Respondents provided between 1–7 cases with an average of 2.9 examples per HW. There were no major differences in the averages of cases shared between the districts and professions. In the following, the cases are analysed according to: 1) type of actors providing feedback; 2) topics and types of feedback; and 3) reaction of HWs to these types of feedback.

### Types of social actors

Table 2 presents the different terms HWs used to describe social actors. Actors have been categorized into nine larger groups and further clustered into five groups of actors: recipients (women who actually received services, usually accompanied by relatives, guardians or husbands), community leaders, the community in general, co-workers, and the spouse and friends of the HW. According to the data in Table 2, recipients of maternal health services feature most frequently in the feedback cases (52%), followed by community leaders (21%) and the other three groups, together representing 27% of the cases.

**Table 2:** Types and frequencies of social actors featuring in the feedback cases (total n = 155).

Terms used by respondents	Subcategory	Cluster of actors	# appearance in the cases
Women (sometimes referred to as clients or patients) coming for antenatal, delivery, and postnatal care, their husbands, guardians and/or accompanying relatives	Women Guardians Husbands	Recipients	81 (52%)
Health Centre Advisory Committee: chairman, chairwoman, treasurer, HCAC members (all referring to the community representatives within the Committee)	HCAC	Community leaders	33 (21%)
Chiefs, village headmen, Traditional Authority (TA), [Ward] councillor, Member of Parliament	Chief		
Pastor, priest, church leader, clergy	Pastor		
Community members, people in the community, men, women in general	Community	Community	17 (11%)
Co-workers: colleagues, matron, in-charge, Health Surveillance Assistant (HSA), hospital attendant	Co-workers	Co-workers	13 (9%)
Husband, wife, fiancé	Spouse of HW	Spouse of HW and friends	11 (7%)
Friends, neighbours	Friends		

Within the recipient group, women who come for their delivery were mentioned most often, followed by guardians, relatives and husbands. Within the group of community leaders, HCAC members and chiefs were mentioned in an almost equal number of cases. The data show the heterogeneity of social actors having regular interactions with HWs on their performance, including spouses and friends in the private sphere and the HCAC as the more formal structure with the mandate to monitor health service delivery and manage complaints. Differences were noted between nurses and clinicians whereby nurses (male and female) shared more cases of feedback from relations nearby (spouses and patients) and clinicians from friends, parents and siblings, often living outside of the community. Chiefs and other community leaders were mentioned in equal numbers by nurses and clinicians. Throughout the paper, the actors will generally be mentioned by their cluster name.

### Topics and types of feedback

The different groups of actors provide feedback on different service delivery issues and in different ways. The different feedback methods, actors and topics are summarized in Table 3 and further discussed in the sections that follow. Interview quotes from the cases are used to illustrate the findings; they are provided in the Quote list in S1 Table. The majority of feedback cases concern topics related to the availability (presence and absence), performance (the right decision and action in cases of complicated deliveries) and interpersonal behaviour of individual HWs. The types of feedback HWs receive in their daily practice could be categorized into individualised (verbal one-on-one compliments and complaints by women or intermediaries) and collective (public buzz and involvement of the HCAC and health authorities) categories.

**Table 3:** Frequencies of cases and actors per type of feedback.

Feedback method	# cases	Main social actors	Main topics
**Individual feedback**			
Direct verbal and non-verbal (material and symbolic rewards) expressions of gratitude	75	Recipients	Skilled birth attendance of normal and complicated deliveries; management of complicated deliveries in the HC; successful referral; effort to finding transport; non-discrimination
Indirect verbal feedback provided by women and transferred through intermediaries (both positive and negative)	48	Community leaders (n = 20); Co-workers (n = 13); Friends and spouse of HW (n = 11); Husbands/guardians of recipients (n = 4)	Unfriendly behaviour; absence during childbirth; late arrival
**Collective feedback**			
Public buzz (mostly negative)	17	Community	Waiting times; accusations of neglect, “not willing to help”; accusation of incapacity or neglect after maternal and child death (delayed referrals etc.)
Discussion in HCAC meetings and reports to health authorities (District Health Office)	15	Community leaders	Maternal and child death; repeated misbehaviour

#### Verbal and non-verbal expressions of gratitude for successful deliveries

Table 3 shows that recipients provide positive feedback during and after successful deliveries directly to the HW with verbal expressions of gratitude, and material and symbolic rewards. The gratitude most often concerns the fact that the HW was available, able and/or willing to help. In some cases, recipients were particularly thankful that the HW did not discriminate against them for not having attended the mandatory antenatal care (ANC) services or for arriving at the health centre when labour had already started (late arrival). One nurse, for example, explains how a post-delivery feedback chat with a woman and her husband reminds her to treat patients equally, regardless of their pre-childbirth situation (e.g. poverty, non-adherence to ANC, late arrival) or regardless of pressure by other women to *not* treat certain women (Quote A in S1 Table). In a few instances, women and guardians thank HWs for their kind and friendly behaviour.

Although expressions of gratitude from recipients appear most frequently in the feedback accounts, a number of respondents indicated that not all women thank for the services received as *“people and cultures are different”* and *“we are not all born the same”*. One nurse estimates that about one in four women (or their relatives and guardians) express their gratitude for a successful normal delivery at the labour ward or before discharge. Other nurses, however, experience that almost all women show some form of appreciation, even if it is just a facial expression. Expressions of gratitude for positive birth outcomes after complicated deliveries and referrals are more frequent and explicit than after normal deliveries. In such cases, women and their relatives almost always come to thank the HW verbally, sometimes multiple times: after the delivery, during the postnatal check-up and on the street. Even in the case of a maternal death, a male nurse was thanked for trying everything possible to save a woman with anaemia by personally travelling to surrounding facilities to find blood.

Material and symbolic rewards for successful deliveries involve women and their relatives offering gifts or food items or asking the HW to name the baby. One nurse experienced being called “doctor” instead of “nurse” as a form of appreciation, and another felt appreciated when a newborn was being presented as her daughter. Such material or symbolic feedback was reported 17 times, most often in cases of successful complicated deliveries conducted at the HC (Quote B in S1 Table), risk-bearing referrals (Quote C in S1 Table) or near-misses, and as an addition to verbal feedback. The respondents, who shared such examples, indicated that they are rewarded in this way occasionally, about once a month.

#### Compliments and complaints via intermediaries

There were no examples of women directly complaining to the HW on the care received. In just two cases, the guardian or husband spoke for the women “on the spot” complaining, for example, about the absence of material (e.g. mattress cover) or the lack of attention from the HW. According to the HWs, disapproval of the HW’s services is sometimes expressed by non-verbal acts, including the absence of comments, and women and guardians leaving the HC without saying goodbye. Most critical feedback or complaints, however, are expressed verbally but indirectly through intermediaries.

The indirect feedback cases mostly concerned critical feedback or complaints, rather than compliments. Table 3 shows that different intermediaries feature in these cases: community leaders, co-workers, the HW’s friends or spouse (Quote D in S1 Table), or the recipient’s husband. Co-workers are auxiliary staff at the HC: ward assistants, maternity home staff, Health Surveillance Assistants (HSAs) or watchmen. HSAs perform home visits where they hear about women’s satisfaction or dissatisfaction with the services at the HC (Quote E in S1 Table). According to respondents, these intermediaries help to overcome women’s fear to speak directly to HWs. Some HWs highly appreciate feedback from co-workers, especially those who *“know the community”* (Quote F in S1 Table).

According to some respondents, HCAC members, often the chairman individually, provides positive as well as negative feedback “on the spot”, immediately after having received a compliment or complaint. Instances were shared where HCAC members question HWs, for example when a HW has been to a meeting in the district and the HC was closed; or the HCAC would come to complain about patients that were not helped or about the lack of drugs. More (male) clinicians talk about the HCAC than nurses and females. This may be explained by the fact that the formal counterpart of the HCAC in the HC is the officer-in-charge. In the study sites, most in-charge positions are filled by (male) clinicians.

#### Public “buzz”

A number of feedback cases (n = 17) revealed that users or communities also have a collective approach to communicate their satisfaction or disapproval of HWs or the HC. Rather than receiving feedback from particular persons, feedback then reaches HWs through conversations in public spaces.

Some positive feedback reaches the HW through chatting with people at the market or when engaging in village activities. A female nurse, for example, mentioned she chats with other women at the central flourmill and a young HW states he receives compliments informally through youth groups in the village. In cases of a near-miss or saved baby, HWs reported being called a hero or a God in the streets. Cases of a negative buzz were also reported with respondents explaining that maternal and child deaths which in the eyes of the community are always preventable, could generate negative feedback, ranging from looks of disapproval in the community to accusations of killing. A nurse presented a case where a woman delivered on the way to the referral hospital; the nurse overheard women talking among each other about “*the nurse that doesn’t know how to work*”. Around the HC, during opening hours as well, HWs report hearing patients grumbling among each other about the long waiting time or unfriendly HWs. While women do this in the maternity home or shelter with each other, husbands are more vocal in public about the quality of services as reported by multiple HWs (Quote G in S1 Table).

#### Involvement of HCAC and health authorities

Both the individual and collective informal feedback methods described so far concerned spontaneous or non-organised feedback actions, unsolicited by either the HW or community structures such as the HCAC. Therefore, the majority of these actions are not linked to local formal accountability mechanisms. The HCAC seems to function as a “hybrid” structure offering, on the one hand, informal verbal feedback on the basis of personal relations between HCAC members and HWs, and, on the other hand, a formal feedback structure that takes up some complaints as a group and sometimes uses its authority to report “serious cases” to district authorities. A few HWs reported that they were occasionally invited to HCAC meetings to discuss complaints. In seven cases, the HCAC used its authority to enforce explanation from the HW by involving the district health authority. Three cases involved maternal or child deaths that were reported to the District Health Office. In four other instances, the chief and the HCAC jointly reported acute problems (striking of health staff) and recurrent misbehaviour (drunkenness, abusive behaviour) of a nurse or clinician to the District Health Office. Most of these processes involved written documentation.

### Effect on health workers

Health workers were asked to indicate how they felt about feedback incidences and how these affected their behaviour towards their work and their patients. Four HWs said that the evaluation and judgement by the community does not influence their work. According to a female nurse and an officer-in-charge, the health outcome itself, the delivery of a healthy baby or failure to do so, is already the main indicator of performance; social feedback does not influence that. On the other extreme, three HWs stated community feedback was their most important source of feedback (Quotes H-J in S1 Table).

HWs described their feelings towards instances of appreciation in different ways: feelings of happiness were most mentioned, followed by feelings of pride and value, confirmation, achievement and self-confidence. Being seen as a professional by patients was valued by one male clinician who said *“as of now*, *I am someone”* after having been hugged by women on the labour ward after a successful delivery. For 18 HWs, instances of negative feedback triggered a range of negative feelings: feeling aggrieved, bad, incompetent and demotivated. In cases of maternal or child death, HWs often felt aggrieved or falsely accused by the community’s comments “*as if I had not done anything*” and “*it is not my fault”*. HWs often attributed death as a result of late or failed referral to users’ neglect of procedures or the poorly functioning health system; community’s complaints were then considered misplaced. But also in cases of successful referral, a HW felt offended when she heard afterwards that she was blamed for not assisting women in the local HC: “*I thought I was doing good to them [by referring]*, *but now they are saying I didn’t assist them”*. Four health workers reported that such cases of rejection made them depressed, afraid to make mistakes, or physically unable to work well, wanting a rest to recover or to leave the job all together.

Most HWs reported that both positive and negative feedback encouraged them to continue in the same way or to work hard, extra, better or more. Two HWs suggested they increased their efforts to avoid coming late and stay throughout childbirth and post-delivery and one modified opening hours of the clinic as a result of feedback. In addition, feedback from social actors raised HWs’ awareness of the importance of the relational aspects of care, and the need to actively engage and “chat” with users, to listen to their concerns and to treat them equally. Negative feedback, specifically around cases of referral, made HWs aware of the need for better sensitisation and education on birth-preparedness. Some realised they needed to intensify their own sensitisation efforts, while others saw this as a task for the government. Some HWs discussed the effect of positive and negative feedback on the community in a broader sense beyond the personal level. Appreciation for good birth outcomes at the facility would trickle down to the community; women who have been treated well would spread the word in their villages, leading to increased overall appreciation of the HC and confidence and trust from the community in delivery care. Similarly, some HWs fear that criticism also gives the HC a poor image.

## Discussion

The primary purpose of this paper was to explore the functioning of informal social accountability relations in a rural setting in Malawi. By analysing accounts of feedback shared by maternal healthcare workers, the paper leads to a better understanding of the types of social pressure HWs face and how they react to it. The types of feedback HWs receive in their daily practice range from individualised (verbal one-on-one compliments and complaints by women or intermediaries) to collective (public buzz) to more formalized processes (involving the HCAC and health authorities). They are part of a larger repertoire of voice mechanisms in which citizens try to obtain more or better services. The cases show the informal character of these processes; most of them are unorganised, spontaneous or unsolicited by either the HW or community structures such as the HCAC. The findings support our assumptions that informal social accountability relies on citizens exerting soft pressure on HWs through expressions of gratitude and approval, appeals to pride and responsibility, combined with tactics to indirectly communicate complaints. Shaming and embarrassment in public is used to some extent.

Informal forms of social feedback, both positive and negative, seem to encourage most HWs to provide more effort and they remind them of the relational and communicative aspects of care. The findings show that informal feedback and accountability has its limitations in that it is inadequate to deal with “serious cases” of abuse, failed referrals and assumed neglect, leading to maternal or child death. Such incidents are complex, related to individual personal factors or based on accountability failures at multiple levels of the health system, beyond the sphere of control of communities and HWs. When informal feedback then evolves around suspicion and blame of individual HWs, it can have a negative effect on patient-provider interpersonal communication and interaction, and on public trust in HCs, even leading to conflict, including violence against HWs [16].

While social accountability is often conceptualised as a formal process or an intervention, this study draws attention to existing accountability relations in the daily life of rural HWs. There are at least two reasons why it is important to pay attention to these relations. First, at the individual level, accountability in the form of feedback and informal sanctions trigger both incentives and disincentives for HWs to maintain or provide respectful care. Second, the presence and influence of an informal accountability web has implications for formal social accountability initiatives.

### Informal social accountability as incentives to perform for rural HWs

Given that the findings generally focused on instances of positive feedback, we are best able to discuss the incentives provided by informal relations, particularly for rural HWs. The HWs in our study often operate on their own or in teams of two as many HCs do not have the required number of five skilled birth attendants. They try to cope with the limited resources they have at their disposal to provide a basic level of acceptable care. During the interviews, they expressed their frustration with the lack of resources and materials, and with the difficulties of being accepted by the community, especially when coming from another district. HWs regularly received advice from family members, spouses, friends and community leaders on “*how to live in the community*”, how to adapt to a different culture, relate well with others and keep up despite difficult circumstances. In the group discussion, HWs argued that such moral guidance reminds them of their mission and ethical standards learned at medical school. We argue that advice and feedback demonstrating public recognition, mental and social support provide a hidden form of moral pressure on HWs to respect social and professional norms such as punctuality, respect and friendliness. In addition, they constitute important non-material sources for socialisation, motivation and retention, crucial for rural HWs as also suggested in a study on Human Resource Incentives in Malawi [45]. Clearly, these types of incentives are necessary for most HWs, but we cannot assume that they work for all HWs, as illustrated by the diverging reactions of HWs and the denial of responsibility that some forms of feedback generate. Whereas this study provides insights into the non-material incentives informal social feedback are likely to generate in individual HWs, we cannot draw firm conclusions about the effect on retention in the long term or on health services or outcomes beyond the individual level. Such changes depend on many other factors, such as the link with formal structures and extrinsic motivation, than just the reactions and behavior of individual HW to social feedback.

### Implications: Linking informal and formal social accountability

While it is important to recognize that daily feedback and advice mechanisms matter for HWs in rural settings, we raise questions as to how they relate to formal social accountability initiatives. If much accountability takes place in the form of daily, informal and hidden interactions, one has to ask to what extent they are inclusive. Social accountability mechanisms that are based on individual interactions run the risk of involving only vocal citizens or those who have the best connections, potentially leading to HWs exclusive or selective responsiveness [7]. One also has to ask whether, why and how informal social accountability relations should be formalized or steered from the outside. For example, informal interactions, such as “having a chat”, or suggestions to “be friendly next time” are difficult to institutionalise and not likely to be influenced by external interventions or formal procedures for citizen feedback [46]. Similarly, formal participatory mechanisms, such as community scorecards, may be less important as channels for influence on service delivery than informal networks and relationships [28]. The formalization of citizen feedback may crowd out effective soft mechanisms or lead to co-optation by HWs [47]. On the other hand, against the potential drawbacks of interpersonal interactions described above, alternative, less individualised options should be provided to ensure a more collective voice directed at the health facility team, rather than to individuals. The role of health facility committees in providing an accountability interface between HWs and the community has been suggested [19] and studied [48] elsewhere. We suggest to further explore this for the HCAC in Malawi that have the potential to structure feedback processes and to represent a collective voice while maintaining effective local norms of engagement.

Linkages to formal accountability mechanisms beyond the local level are also needed to investigate and react to cases of abuse and disrespect. As Tembo (2013) suggests, *“the incremental making and institutionalisation of informal rules*, *when reinforced with the more formalised procedural accountability*, *can build accountability relationships that work”* (Tembo, 2013: 44[49]). From our study, two key considerations emerge. First, we emphasize the need for an analysis of the network of accountability relations in a given context prior to the design of participatory or community monitoring interventions by external programmes or aid donors. In our study district, accountability relations blur traditional boundaries like public/private (friends and spouses of HWs as promoters of moral responsibility), personal/professional (some co-workers and HSAs are considered friends or personal informants and more important than the official facility manager) and informal/formal (HCAC members acting as individuals and as a formal structure with procedures). HWs are part of health facility teams, organisations and communities and their accountabilities constantly move between organisational, professional and societal boundaries [14]. Each actor probably draws on a form of legitimacy, authority and credibility to influence HWs’ receptivity to community concerns or behaviour [7]. A systematic exploration of actors, interactions and influence tactics, some of which provide opportunities for change, may contribute to more realistic expectations regarding participants’ incentives and influence in these programmes, also in the long run [13,50]. Such an exploration may also identify how temporary external projects could develop strategies to shift unconstructive to constructive accountability relations between communities and HWs [28] and how local alliances, including representatives from both groups, could be supported to advocate for changes in the health system [51].

Second, the emergence of constructive accountability relations, whether informal or formal, remains largely theoretical when HWs, communities and community structures are not given the authority and the resources to fulfil their mandate to provide and monitor maternal health services [52]. Practical examples to support sub-national accountability networks are devolved budgets and authority to districts and facilities to make changes in the provision of care to enhance quality [52]. Also, pre- and in-service training of HWs in the field of ethics, social and cultural competencies and participatory techniques, and the integration of incentives for person-centredness in performance assessments could enhance professional and respectful behaviour as well as constructive accountability relations [53].

Finally, our study suggests some broader lessons for the field of accountability, governance and health systems beyond social accountability as a sub-field. In the introduction, we suggested that informal accountability relations form a substitute for weak formal social and bureaucratic accountability mechanisms. We suggest that informal social accountability exists regardless of the presence or strength of formal social, bureaucratic or judicial accountability mechanisms. As Therkildsen (2014) suggests, a health system *“is not a bureaucratic machine but a configuration of social processes”* [54]. In general, insufficient attention has been paid to how informal relations work *de facto*, also in contexts where *de jure* accountability structures are well developed and implemented [47,55].

### Strengths and limitations

The collection of case stories of HWs helped to capture the lived experiences of participants. It generated a rich map of social feedback relations and it showed the variety of perspectives on, and reactions to, feedback relations. The results describe the informal accountability network of maternal health care workers in a typical rural district. Service provision conditions and community-provider relations may be similar in other rural Malawian districts and lessons may be transferable to other rural areas, even outside of Malawi. The findings may, however, not be transferable to health facilities and providers in urban districts. We hypothesise that HWs in urban areas are less remote from other accountability mechanisms, such as peer and supervisor feedback, and less dependent on their social environment to get inputs to steer their performance, motivation and retention. Further research should clarify this point.

We accumulated data on a relatively large number of individual HWs’ relations, but the open character of the interviews did not allow for group comparison. Additional research, in particular quantitative approaches to social network analysis, could offer interesting insights into structures of communication and accountability in local health systems and differentiate between groups, based on, for example, profession, gender, age and work experience.

One of the risks of narrative interviews is that informants try to present themselves in a favourable light [56]. In addition, the topic of accountability can be sensitive as it may be interpreted as being related to personal or clinical failure, a lack of responsibility and poor performance. Participants may perceive researchers as evaluators and, hence, provide socially desirable answers. We made efforts to deal with potential discomfort of participants related to the sharing of personal experiences by implementing a strong consent process and by applying a conversational and appreciative style of interviewing. We acknowledge, however, that the topic and interview style might have led to a bias towards the sharing of cases of positive feedback and the presentation of negative feedback as a positive phenomenon. For example, from other research in Malawi, we know that community-provider relations can be conflictual and even violent [57], but such examples barely surfaced in our interviews.

Internal reliability was improved by the use of multiple researchers in the field and at the analysis stage. The coding schemes were jointly developed and tested by three researchers. Participants’ accounts were coded by two researchers who aimed to reach a consensus on the interpretation of each case.

## Conclusion

While social accountability is often conceptualised as a formal process or an intervention, this study shows that existing daily relations of feedback and informal sanctions play an important role in promoting or maintaining respectful care. This paper argues for revisiting the assumptions of social accountability. In the development and evaluation of social accountability interventions, it would be useful to analyse these spontaneous types of feedback in addition to the solicited ones through formal interventions. In addition, given the suggested importance of a web of social accountability relations for health worker performance, it is important to understand how health workers perceive themselves and their responsibilities vis-à-vis their social environment and to identify ways in which their interaction and relations with communities and other professionals can be enhanced.

## Supporting information

S1 Table Quote list. (DOCX)
