# Supplementary material for: Informal social accountability in maternal health service delivery: A study in Northern Malawi
Source: PLoS One. 2018 Apr 11;13(4):e0195671. doi: 10.1371/journal.pone.0195671 (PMC5895061; doi:10.1371/journal.pone.0195671)
Supplement: S1 Table — (DOCX) [file pone.0195671.s001.docx]

**Supporting file 1. Table**

| **Verbal and non-verbal expressions of gratitude for successful deliveries** | |
| --- | --- |
| Quote A  Female nurse  16 years in HC | *‘She delivered some twins. So the husband was very happy outside and he came with some pumpkins and a bundle of fire wood. They brought it to my home, said thank you very much, I know you did a great job, because my wife was shy to come because she didn’t have a nice cloth to come to antenatal. I said ‘no that’s not true, anybody can come, we welcome her’. So he was very delighted that we delivered the wife, with two babies, all in good condition…But the community said nobody should be attended if one doesn’t attend the antenatal clinic, she should be penalized. I said ‘no, we don’t, as long as she has come and delivered at the hospital’. I felt that is good, sometimes to be patient..don’t just react when the patient comes in who hasn’t any goods, because we know that behind it, there must be a reason. Because at last she told me ‘I didn’t have nice clothes for antenatal’. So if we just come here, shouting at the mothers, it’s not good’.* |
| Quote B  Female nurse  7 months in HC | *‘It was a time, there was a certain girl just down there, she was short…then I said ‘ah the way I can see this girl, I should send her to the district hospital because this is a small hospital’. Then there was no transport, and the mother pleaded ‘oh nurse can you try?’. Then I could assist the woman, I gave her a time…then she progressed and then she delivered a bouncing baby boy. Then they, the mother in law was very happy and then she went to the grocery and said ‘oh can you have a glass of fanta because you have done a very good job there’*. |
| Quote C  Male clinician  1 year in HC | *‘They are not many [appreciation]…but there was a time we had a woman whom we suspected had a rupture of uterus and there was no fuel, government fuel, there was no ambulance for government. We consulted so many people including the MP so that he can assist in transport, so that we can refer her to another facility…In the end the traditional authority, the T/A gave us a car and that patient was referred to another facility in which she survived. If we had delayed, that patient could have died and the community, ah they applauded me, the community… show appreciation upon me, the husband of that lady he gave me a chicken’.* |
| **Compliments and complaints via intermediaries** | |
| Quote D  Female nurse  2 years in HC | *‘My husband is a part of the community as well. Once I went to Mzuzu, there was a meeting, so when coming back…my husband was saying, “ah some people came here, they were voicing…saying ah these people are not assisting us, they have all gone out”, so yah’.* |
| Quote E  Female nurse  3 years in HC | *‘Yah they [HSA] do comment. When sometimes you provided good care to the patient, when they go back to their villages, they do talk…so if they talk maybe…they do appreciate you. The HSAs are there and they come back and tell you you are doing a good job. These people are saying this this this this…If you have done something wrong, they also discuss there in the village, the HSAs also come and tell you that, “you have to change”, “I think there is a problem here, you have to take care of these people”, whatsoever yeah’.* |
| Quote F  Female nurse  10 years in HC | *‘She [colleague nurse], also helps me because she knows this community, and the people around this hospital, so she usually teaches me how we welcome patients here, how we are supposed to deliver services to these people, while coming from the community’.* |
| **Public buzz** | |
| Quote G  Female nurse  2 years in HC | *‘The husbands of the women said ‘they are not helping us’. [The husbands] are just voicing, moving on the road and talking. I heard from other people that “ah, people are complaining that you have just let them, those husbands”’.* |
| **Effect on health workers** | |
| Quote H Female nurse  14 years in HC | *‘…We stay with our clients’ in the village here, the government is far away, they will just appraise on papers [..] but the women, they do see us.”* |
| Quote I  Male clinician  1 year in HC | *‘The supervisors, eh…they don’t talk most times, they only talk about the protocols, the health protocols. So I value most the feedback from the patients, not the supervisors’.* |
| Quote J  Male clinician in group discussion | *‘[...] but when this guardian comes to me in a polite manner, [...] I think that is much more powerful than when you are hearing from the [HCAC] chair person, because hereby it is his job. [...] So it will be good other than using the authorities only’.* |
